# The potential role of Osteopontin in the maintenance of commensal bacteria homeostasis in the intestine

**DOI:** 10.1371/journal.pone.0173629

**Published:** 2017-03-15

**Authors:** Koyu Ito, Akira Nakajima, Yuji Fukushima, Keiichiro Suzuki, Keiko Sakamoto, Yoko Hamazaki, Kouetsu Ogasawara, Nagahiro Minato, Masakazu Hattori

**Affiliations:** 1 Center for Innovation in Immunoregulative Technology and Therapeutics, Graduate School of Medicine, Kyoto University, Yoshida Konoe-cho, Sakyo-ku, Kyoto, Japan; 2 Department of Immunobiology, Institute of Development, Ageing, and Cancer, Tohoku University, Aoba-ku, Sendai, Miyagi, Japan; 3 Department of Immunology and Cell Biology, Graduate School of Medicine, Kyoto University, Yoshida Konoe-cho, Sakyo-ku, Kyoto, Japan; Forsyth Institute, UNITED STATES

## Abstract

Osteopontin (Opn), a multifunctional extracellular matrix protein, is implicated in the pathogenesis of various inflammatory disorders. Under physiologic conditions, its expression is restricted to certain tissues including bone and kidney tubule. However, cellular activation during disease development induces Opn expression in various immune cells. In this study, using Opn-EGFP knock-in (KI) mice we found that CD8α^+^ T cells in the intestinal tissues, including Peyer’s patch, lamina propria and epithelium, express Opn under steady state conditions. Therefore, we examined the role of Opn-expressing CD8α^+^ T cells in intestinal homeostasis. Interestingly, Opn knockout (KO) mice had altered fecal microflora concordant with a reduction of TCRγδ^+^ intraepithelial lymphocytes (IELs). Consistent with this result, both treatment with anti-Opn blocking antibody and deficiency of Opn resulted in decreased survival of TCRγδ^+^ and TCRαβ^+^ IELs. This data suggests that a possibility that Opn may function as a survival factor for IELs in the intestinal tissue. Collectively, these data suggest the possibility that Opn might regulate the homeostasis of intestinal microflora through maintenance of TCRγδ^+^ IELs, possibly by support of IEL survival.

## Introduction

Osteopontin (Opn), a multifunctional extracellular matrix protein, contains at least two distinct cell-binding domains; Arg^159^-Gly-Asp^161^ (RGD), which binds to the RGD-recognizing integrin αvβ3, and Ser^162^-Val-Val-Tyr-Gly-Leu-Arg^168^ (SVVYGLR), which binds to α4 and α9 integrins. Under physiologic conditions, expression of Opn is known to be restricted to tissues such as bone and kidney. In these organs, Opn has been shown to be involved in various physiological functions including biomineralization of bone [1,2] and the regulation of renal crystal formation [3]. In contrast, Opn is upregulated in inflamed and injured tissues, and is implicated in the pathogenesis of various inflammatory disorders [4], tissue remodeling [5], wound healing [6], tumor invasion [7], and metastasis [8].

Of note, Opn was shown by immunohistochemistry to be distributed on epithelial cells and plasma cells in normal human colon tissue [9]. Several groups reported that Opn is involved in inflammatory bowel diseases (IBDs) including Crohn’s disease (CD) and ulcerative colitis (UC), which are caused by excessive responses to commensal microbiota and other intestinal antigens [10]. Further, colon tissues from CD and UC showed upregulation of Opn [9] and Opn-deficient mice were resistant to 2, 4, 6-trinitro benzene sulfonic acid (TNBS) [11] and dextran sulfate sodium (DSS)-induced colitis [12], which are the models for CD and UC, respectively. During disease development, Opn was markedly upregulated in various immune cells, such as lymphocytes and DCs. Among these, CD103^-^ dendritic cells highly express Opn and transfer of these cells induces severe acute colitis concordantly with increases of IL-17 and IFN-γ-producing CD4^+^ T cells [13]. As described above, the role of Opn in inflamed conditions has been demonstrated, whereas the significance of Opn in the normal gut remains unclear. Intestinal tissue is continuously exposed to antigens from microbiota, and Opn expression is increased in response to some infections, such as pneumococcal pneumonia and dengue virus [14,15]. Therefore, it has been speculated that Opn might be induced in immune cells following exposure to antigens from these microbes.

Commensal microbiota are critical for maintenance of the intestinal immune system [16] and the intestinal environment is controlled by 1) immunoglobulin A (IgA) from B cells present in the gut associated lymphoid tissue (GALT), particularly in Peyer’s patch (PP) and lamina propria (LP) [16], and 2) antimicrobial peptides produced by intraepithelial lymphocytes (IEL) and epithelial cells [16,17]. Microbes bind to toll-like receptors (TLRs) on intestinal epithelial cells leading to the production of B cell activating factor (BAFF) and a proliferation-inducing ligand (APRIL), which promote IgA-producing B cell generation and subsequent plasma cell differentiation. Consequently, IgA is secreted into the lumen and regulates microbiota composition [18]. On the other hand, recognition of microbes by epithelial cells through MyD88-pathways is required for production of antimicrobial peptides from IELs [17]. These antimicrobial peptides kill bacteria by various mechanisms, such as inhibition of bacteria growth [19,20], interference with metabolism [21–23], and generation of reactive nitrogen species and reactive oxygen species [24]. Deficiency and/or dysfunction of IgA or IELs result in alteration of the bacterial community and increased entry of bacteria into the intestinal epithelial tissue [17,25].

To clarify the role of Opn in the normal intestinal tissues, we first used reporter mice in which the Opn gene was replaced by EGFP (EGFP-Opn knock-in mice) to monitor Opn expression [26]. We found that in normal mice, CD8α^+^ T cells in the PP, LP, and intestinal epithelium expressed Opn. Notably, in Opn-deficient mice, there was a reduction of frequency and number in TCRγδ^+^ IELs and the microbiota of fecal samples was drastically altered as compared with WT mice. Thus, our data suggest the possibility that Opn might contribute to maintenance of homeostasis of intestinal commensal bacteria, possibly by TCRγδ^+^ IEL survival.

## Materials and methods

### Ethics statement

Mice were maintained under specific pathogen-free conditions at the Center for Experimental Animals of Kyoto University, and all procedures were performed according to the protocols approved by the Institutional Committee for Use and Care of Laboratory Animals of Kyoto University, which was granted by Kyoto University Ethics Review Board (MedKyo14064), and the Guide for Care and Use of Laboratory Animals published by the U.S. National Institutes of Health (NIH publication 85–23, revised 1996). For collection of tissue samples, mice were sacrificed by cervical dislocation. All efforts were made to minimize suffering.

### Mice

C57BL/6 mice and Opn Knock out (Opn KO) mice were purchased from Japan SLC and Jackson Laboratory, respectively. EGFP-Opn knock-in (KI) mice were generated as described previously [26]. These mice were bred and maintained at least three generations at the Center for Experimental Animals of Kyoto University.

### Antibodies and reagents

PE anti-CD3, Pacific Blue anti-CD8α, biotin-conjugated anti-CD8β, PE Cy7 anti-CD4, APC Cy7 anti-CD62L, APC anti-CD103, PerCP Cy5.5 anti-TCRγδ, PE Cy7 anti-TCRβ, APC Cy7-B220, PerCP Cy5.5 anti-B220, FITC-EpCAM, PE Cy7 anti-CD11b, APC anti-F4/80, biotin-conjugated anti-IgA antibodies were purchased from BioLegend. Streptavidin-PE was purchased from BD Biosciences.

### Preparation of cells from intestinal epithelium and lamina propria

Small intestines were collected and Peyer’s Patches and fat tissue were carefully excised. After pushing out the intestinal contents, the intestine was opened longitudinally. Then, pieces of intestine were stirred with 5% FCS, 5 mM EDTA, and 1 mM DTT-containing PBS for 30 min at 37°C. Intestinal tissues were placed on a cell strainer, and the cells that passed through the strainer were collected. Remnant tissues were collected, further cut into small pieces, and then stirred with 0.15% collagenase D (Roche) / RPMI for 40 min at 37°C. After stirring, lamina propria cells were collected in the supernatant. This collagenase digestion step was repeated twice. For isolation of lymphocytes and epithelial cells, cell suspensions were separated with a 40/70% Percoll gradient and the intermediate layer and top layer were collected for analysis of lymphocytes and epithelial cells, respectively.

### Flow cytometric analysis

Lymphocytes from lamina propria (LP) and intraepithelium were isolated as described above. Peyer’s patches (PP), inguinal lymph nodes (iLN), mesenteric lymph nodes (mLN), and spleen (Spl) were isolated, and erythrocytes were lysed with ACK lysis buffer (155 mM NH_4_Cl, 10 mM KHCO_3_, 1 mM EDTA). Single cell suspensions were stained with primary antibodies for CD3, CD8α, CD8β, CD62L, CD103, TCRγδ, TCRβ, B220, CD11b, and F4/80. To evaluate IgA-producing cells, cells were stained with biotin-conjugated anti IgA antibody, followed by Streptavidin-PE. In some experiments, IELs (CD3^+^CD8α^+^TCRβ^+^ or TCRγδ^+^ cells) and intestinal epithelial cells (CD103^-^EpCAM^+^ cells) were sorted by a FACS Aria II or III (BD Biosciences) from isolated intestinal epithelium. All analyses were performed on a FACS Canto II (BD Biosciences) with FlowJo Software (Tree Star).

### Evaluation of IgA-coated fecal bacteria

Flow cytometric analysis of bacteria was performed as described previously [27] with some modification. In brief, feces were collected and mashed with syringe plunger in PBS. After filtration with 70 μm nylon mesh, bacteria were separated by 20% / 80% Percoll gradient. After washing with PBS, bacteria were stained with biotin-conjugated anti-IgA antibody as described above. For detection of bacteria, feces were stained with Propidium Iodide (PI).

### Histology

Intestinal tissues of KI mice were obtained and fixed with 2% paraformaldehyde for 2 hours at room temperature, and then tissues were placed in 30% sucrose overnight at 4°C. Tissues were then embedded with OCT compound and frozen by liquid nitrogen. After sectioning (6 μm) and drying, sections were blocked with 1% BSA/PBS, and then tissues were incubated with a primary antibody against CD8α for 1 hour at room temperature, followed by Cy3-conjugated anti-rat IgG antibody. Finally, tissues were stained with APC-anti B220 antibody for 1 hour and sections were analyzed by laser confocal microscopy (LSM710, Zeiss).

### Fecal DNA preparation and analysis of fecal microbiota (Illumina MiSeq)

Feces were collected and genome DNA was extracted using QIAamp DNA stool mini kit (QIAGEN). Microbiome analysis was conducted by Illumina MiSeq. In brief, the V3 and V4 region of 16S rRNA was amplified by PCR and the amplicon was cleaned using the QIAquick Gel Extraction kit (QIAGEN). Cleaned amplicon was then used to prepare a library by limited cycle PCR, which was then d on an Illumina MiSeq (Illumina).

### RNA extraction and quantitative real-time PCR analysis

TCRγδ^+^ IELs, defined as CD3^+^CD8α^+^ TCRγδ^+^, and Epithelial cells, defined as EpCAM^+^CD103^-^ from Opn KO and WT mice were sorted as described above and were lysed with TRIzol reagent (Invitrogen) to extract total RNA. For quantitative PCR analysis, extracted RNAs were utilized to synthesize cDNA using a first strand cDNA synthesis kit (Roche). Real-time PCR was performed using LightCycler-FastStart DNA Master SYBR Green I Systems (Roche). All specific primers used in this study are shown in S1 Table. Expression levels were determined using the -⊿⊿Ct method of real-time PCR. Data were normalized against the expression of cyclophillin.

### Survival assay

Each IEL subset (TCRαβ and TCRγδ) was sorted from 6–10 wk old female Opn KO and WT mice as described above. Twenty-thousand cells of each type were seeded into 96-well round-bottom plate (FALCON) in 10% FCS/RPMI. Cells were cultured for 12 hours in the presence of anti-murine Opn antibody (35B6) [3] or control antibody (10 μg/ml). Surviving cells were counted using a conventional counting method with Trypan Blue and Cell Titer Glo (Promega) according to the manufactures’ instructions.

### Statistical analyses

All data are presented as the mean ±S.E.M or ±S.D as described in Figure legend. Significance of the difference between two groups was determined using Student’s t-test. * and ** denote p<0.05 and p<0.01, respectively, against control. N.S., not significant.

## Results

### Intestinal CD8^+^ T cells express Opn

**Fig 1:**
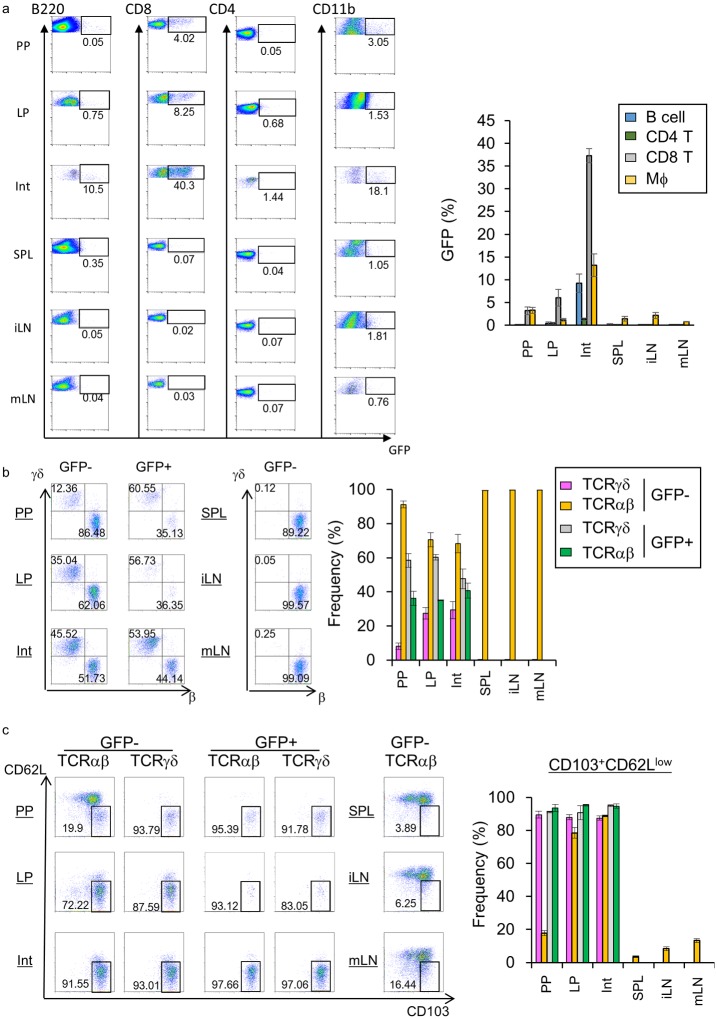
Expression of Opn in specific CD8^+^ T cell subsets in the intestine, but not in other secondary lymphoid organs. Six-week-old female EGFP-Opn mice were analyzed by flow cytometry. Cells obtained from Peyer’s patches (PP), lamina propria (LP), intestinal epithelium (Int), spleen (SPL), inguinal (iLN), and mesenteric lymph nodes (mLN) were stained for detection of (a) CD4 (CD3^+^CD4^+^), CD8 T cells (CD3^+^CD8α^+^), B cells (B220^+^), and macrophages (CD11b^+^) (b) TCRαβ or TCRγδ^+^ cells in CD3^+^CD8α^+^ cells (c) CD103^+^CD62L^low^ resident memory-type CD8 T cells. Graphs show (a) GFP^+^ cells, (b) TCRαβ^+^ and TCRγδ^+^, and (c) CD103^+^CD62L^low^ TCRαβ^+^ or TCRγδ^+^ cells in GFP^+^ or GFP^-^ populations. Bars indicate ±S.E.M (n = 3~5). Data are representative of two independent experiments.

**Fig 2:**
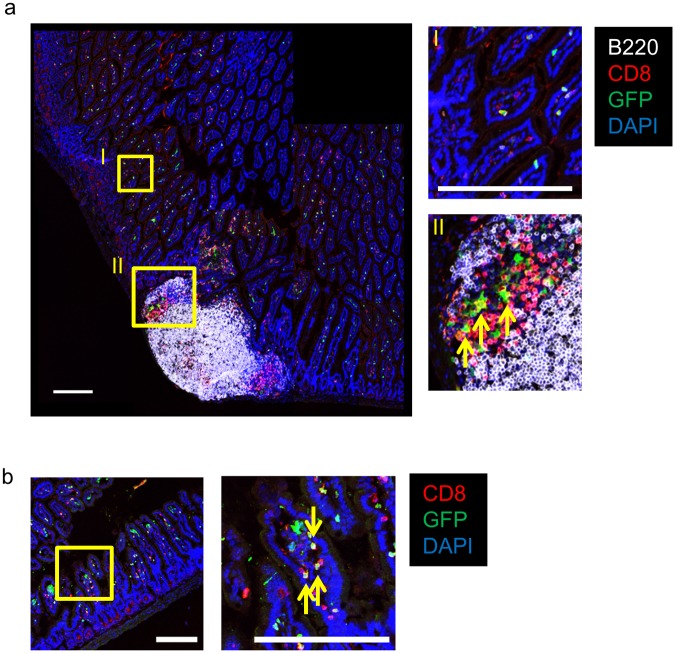
Localization of Opn-expressing CD8 T cells in the intestine. Representative pictures of longitudinal section of intestine (a) including PP and (b) distal to PP obtained from EGFP-Opn mice and stained with B220 (white), CD8α (red), DAPI (blue). Inset shows magnified images. Arrows indicate GFP-expressing CD8α^+^ cells. Scale bars indicate 200 μm. The photograph is representative of 5 mice.

In a previous report Opn was detected in epithelial cells and plasma cells in the normal colon [9]. Therefore, we first identified the source of Opn in the intestinal tissue using Opn-reporter (EGFP-Opn knock-in (KI)) mice, which harbor the EGFP construct in the place of Opn [26]. Using hemizygous KI mice, we performed flow cytometric analysis to examine the source of Opn. Of note, CD8α^+^ T cells in the intestine, including those in Peyer’s Patches (PPs), lamina propria (LP), and intestinal epithelial lymphocytes (IELs), expressed GFP (Fig 1a). We also examined the expression of Opn on plasma cells and epithelial cells in the intestinal tissue, as reported previously; however, we did not observe Opn expression on epithelial cells (S1 Fig). As Opn has been reported to be expressed on activated T cells [26,28], we further characterized these cells. Interestingly, in GFP^+^ CD8α^+^ T cells of the PP and LP, TCRγδ^+^ cells were the majority (Fig 1b). In the intestinal epithelium, GFP^+^TCRγδ^+^ cells were equally exist as compared with TCRαβ^+^ cells (Fig 1b). In contrast, in the GFP^-^ CD8α^+^ T cell population, TCRαβ^+^ cells were dominant in the PP, LP, and IEL (Fig 1b). Almost none of the GFP^+^ TCRγδ^+^ cells in the epithelium expressed CD8β, whereas CD8α^+^ T cells in the spleen expressed CD8β (S2 Fig), indicating that TCRγδ^+^ IELs are CD8αα IEL and not CD8αβ^+^ T cells immigrated from the peripheral lymphoid organs. In addition, these GFP^+^ CD8α^+^ T cells were highly enriched in the CD103^+^CD62L^low^ fraction regardless of TCR expression (Fig 1c), indicating that these GFP^+^ CD8α^+^ T cells were activated. In a previous report, CD103^+^CD62L^low^CD69^+^ CD8α^+^ T cells were shown to be generated at the site of viral or bacterial infection and retained until secondary infection [29,30]. These CD103^+^CD62L^low^CD69^+^CD8α^+^ T cells are referred to as resident memory T cells (Trm cells), and are able to intercept and eradicate pathogens immediately at sites of infections (skin or intestinal mucosa) [29,31]. As the Trm were reported to be localized to the non-lymphoid tissue [29], we also assessed localization of GFP-expressing CD8α T cells. Intestinal tissues of KI mice were immunostained for CD8α and B220 and analyzed by laser confocal microscopy. We found that GFP-expressing CD8α T cells were broadly distributed in the intestine (Fig 2a and 2b), while in the PP, GFP^+^ CD8α^+^ T cells were localized to the T cell zone.

### Opn deficiency affects intestinal bacterial communities

**Fig 3:**
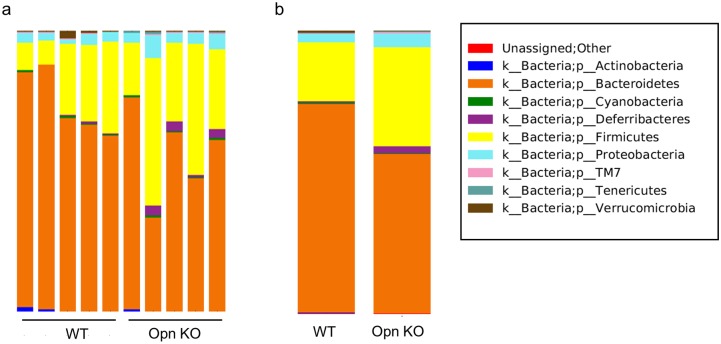
Alteration of fecal bacterial communities in Opn KO mice. Eight-week-old female Opn KO and WT mice were co-housed for 3 weeks prior to collection of feces and extraction of DNA. These mice were bred and maintained in the same facility for at least three generations. Illumina MiSeq analysis of 16S rRNA was then performed. Data are from (a) each sample and (b) the average of the groups. Data was normalized against common bacterial 16S rRNA expression and is presented as a ratio to the value obtained in WT mice (n = 5, per group).

Intestinal tissues such as Peyer’s patches and epithelia are critical sites for the maintenance of intestinal microbiota through production of IgA and antimicrobial peptides, respectively [16]. To examine whether Opn-producing cells play a role in the homeostasis of commensal microflora, we examined the composition of intestinal bacteria using Illumina MiSeq analysis of DNA extracted from fecal samples collected from co-housed WT and Opn KO mice. In order to insure uniform exposure to same condition, these mice were maintained for at least three generations in the same facility. We found that *Bacteroidetes* (WT 49500.2 ± 3092.8 vs KO 38587.2 ± 4731.1) were reduced whereas *Firmicutes* (WT 13969.8 ± 3357.2 vs KO 24107.2 ± 4536.7), *Deferribacteres* (WT 291.4 ± 90.8 vs KO 1600.4 ± 524.2), *Proteobacteria* (WT 1964.6 ± 238.2 vs KO 3337.4 ± 669.6), and *Tenericutes* (WT 68.6 ± 12.7 vs 298.6 ± 111.0) were increased in fecal samples of Opn KO mice (Fig 3 and S2 Table), suggesting that deficient of Opn affected intestinal bacterial communities. In spite of co-housing, Opn KO mice showed alterations in fecal microflora, suggesting that Opn exerts a regulatory mechanism for controlling microflora.

### Opn is not essential for generation of IgA-producing B cells

**Fig 4:**
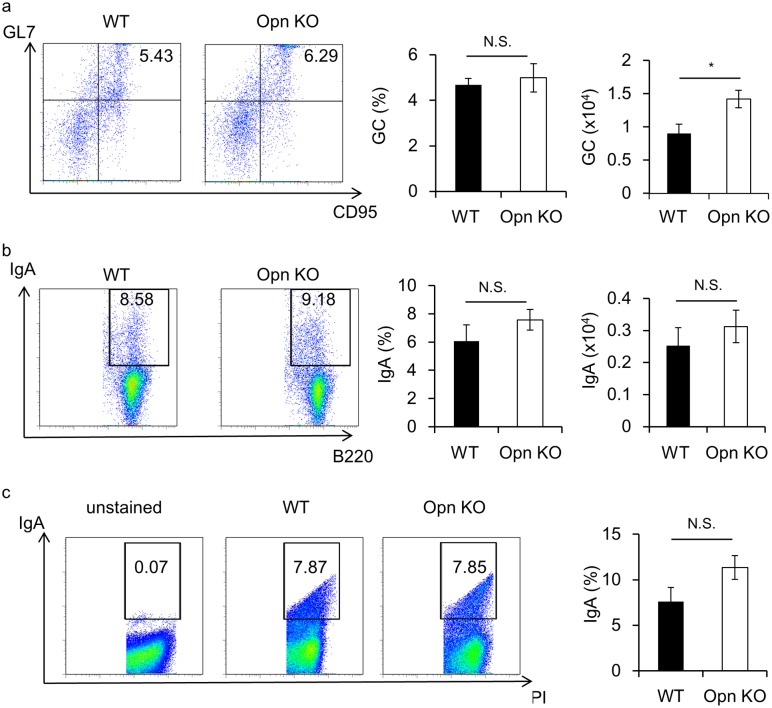
Opn is dispensable for germinal center generation and IgA-producing B cells in PPs. Eight-week-old female Opn KO and WT mice were cohoused for 3 weeks as described for Fig 3. Cells obtained from PPs were analyzed for detection of (a) GC B cell (GL7^+^CD95^+^ B220^+^ cells) and (b) IgA-producing B cells. Graphs show frequency and numbers of GC and IgA^+^ cells gated on B220^+^ cells. (c) Analysis of IgA-coating bacteria in the feces. Bacteria isolated from feces were stained with IgA and PI. PI^+^ events were considered to be bacteria. Graph shows the frequency of IgA^+^ cells with gating on PI^+^ cells. Bars indicate mean ±S.E.M. (n = 5; per group). Data are representative of four independent experiments.

Intestinal microflora is regulated by 1) functional IgA production in the PP and LP and 2) antimicrobial peptide production by IELs and epithelial cells [16,17]. It has been reported that binding of Opn to B cells is crucial for their survival and that Opn-producing CD4^+^ T cells play a role in the development of the spontaneous germinal center (GC), and subsequent production of autoantibodies [26]. To evaluate the molecular mechanisms underlying the role of Opn in the regulation of microflora, we first examined whether Opn plays any role in generation of IgA-producing cells in the PP, which is the inductive site of IgA-producing B cells (Fig 4a and 4b). We observed that in Opn KO mice the total GC number was increased as compared with WT mice (Fig 4a). Of note, IgA-producing B cells were not affected in the PP of Opn KO mice as compared with WT mice (Fig 4b), suggesting that Opn-producing cells in the PP are dispensable for the differentiation of IgA-producing B cells. To further examine the function of Opn in IgA production, we investigated the binding affinity of IgA to bacteria in Opn KO mice (Fig 4c). Consistent with Fig 4a and 4b, the frequency of IgA-coated bacteria in feces of Opn KO mice was comparable to that of WT mice (Fig 4c). These results confirm that Opn is not essential for production of functional IgA.

### Reduction of TCRγδ^+^ T cells in the IEL of Opn KO mice

**Fig 5:**
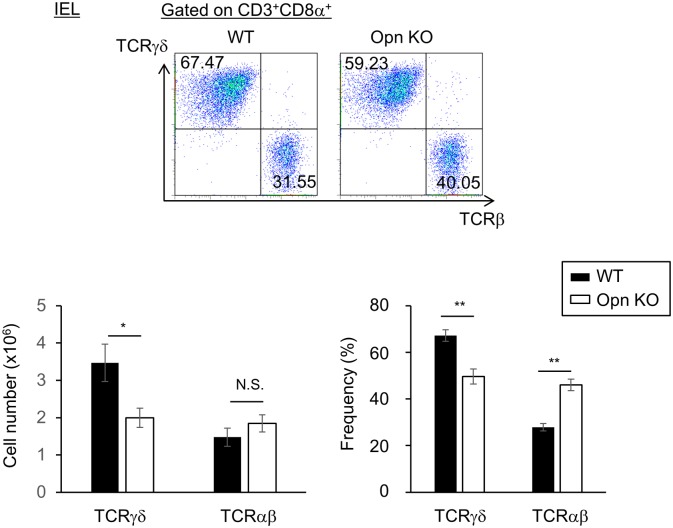
Maintenance of TCRγδ^+^ IELs in the intestinal epithelium by Opn. Analysis of TCRγδ^+^ and TCRαβ^+^ cells in Opn KO and WT mice. Opn KO and WT mice were cohoused as described for Fig 3 and the number and frequency of CD3^+^ CD8α^+^TCRγδ^+^ T cells and CD3^+^ CD8α^+^TCRαβ^+^ T cells in the IEL were analyzed by flow cytometric analysis. (top) The representative dot plots of FACS analysis gated on CD3^+^ CD8α^+^. Graph shows the mean of (lower left) absolute numbers and (lower right) frequency of TCRγδ^+^ and TCRαβ^+^ cells. Bars indicate ±S.E.M. (n = 4~5; per group). Data are representative of four independent experiments.

Antimicrobial peptides are produced by TCRγδ^+^ IELs and intestinal epithelial cells, resulting in the maintenance of commensal microbiota. Thus, we next attempted to determine the significance of Opn expression in TCRγδ^+^ IELs. To this end, we first examined whether deficiency of Opn affects the frequency of these cells. Eight-week-old, female Opn KO and WT mice were co-housed for three weeks prior to the analysis. Interestingly, Opn KO mice showed decreases in the frequency and number of CD8α^+^TCRγδ^+^ IELs compared to WT mice (Fig 5), suggesting the possibility that maintenance of CD8α^+^TCRγδ^+^ IELs in the intestine is disrupted by the loss of Opn.

**Fig 6:**
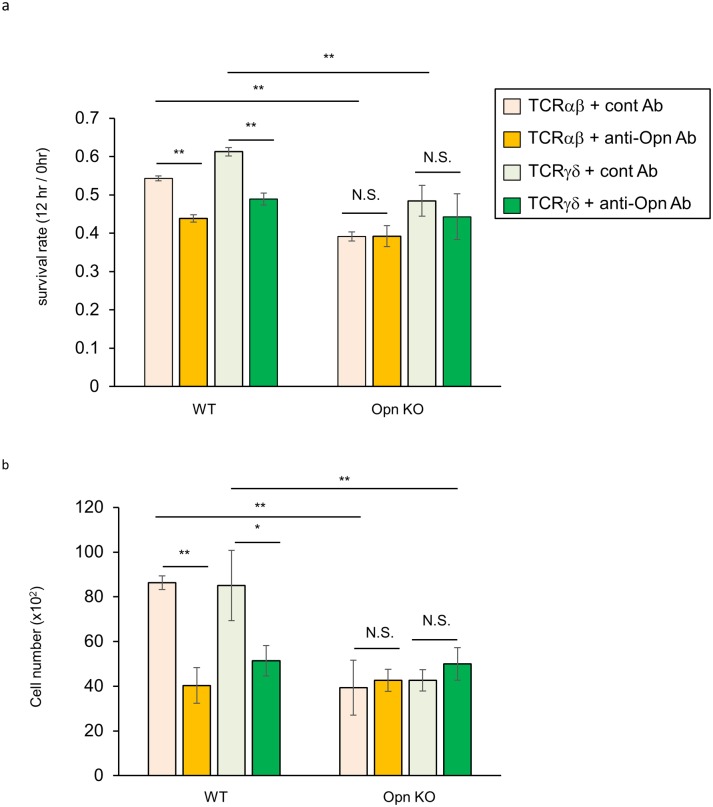
Secreted Opn functions as a survival factor for IELs. *In vitro* survival assay. TCRαβ^+^ or TCRγδ^+^ cells were sorted from WT and Opn KO mice and each of 2 x10^4^ cells was cultured for 12 hours in the presence of 10 μg/ml of anti-Opn blocking antibody (35B6) or control antibody. After cultivation, live cells were counted by (a) Cell Titer Glo and (b) conventional cell counting methods (see Materials and methods). Results were normalized to the values at 0 hours. The graphs show the means from triplicate wells. Bars indicate ±S.D. Data is representative of four independent experiments.

The binding of Opn to activated T cells induced prolonged survival and exacerbation of EAE [32]. Therefore, to examine whether Opn contributes to IEL survival, we sorted TCRαβ^+^ and TCRγδ^+^ IELs from WT and Opn KO mice and cells were cultured in the presence of anti-Opn blocking antibody (35B6) [3] for 12 hours. As shown in Fig 6, treatment with anti-Opn antibody inhibited the survival of TCRαβ^+^ and TCRγδ^+^ IELs. In addition, the inhibitory effect of anti-Opn antibody was comparable with that of Opn KO mice, suggesting that the survival effect of Opn is evident both in TCRαβ^+^ and TCRγδ^+^ IELs.

### Expression profiles of antimicrobial proteins on TCRγδ^+^ IELs and epithelial cells between Opn KO mice and WT mice

**Fig 7:**
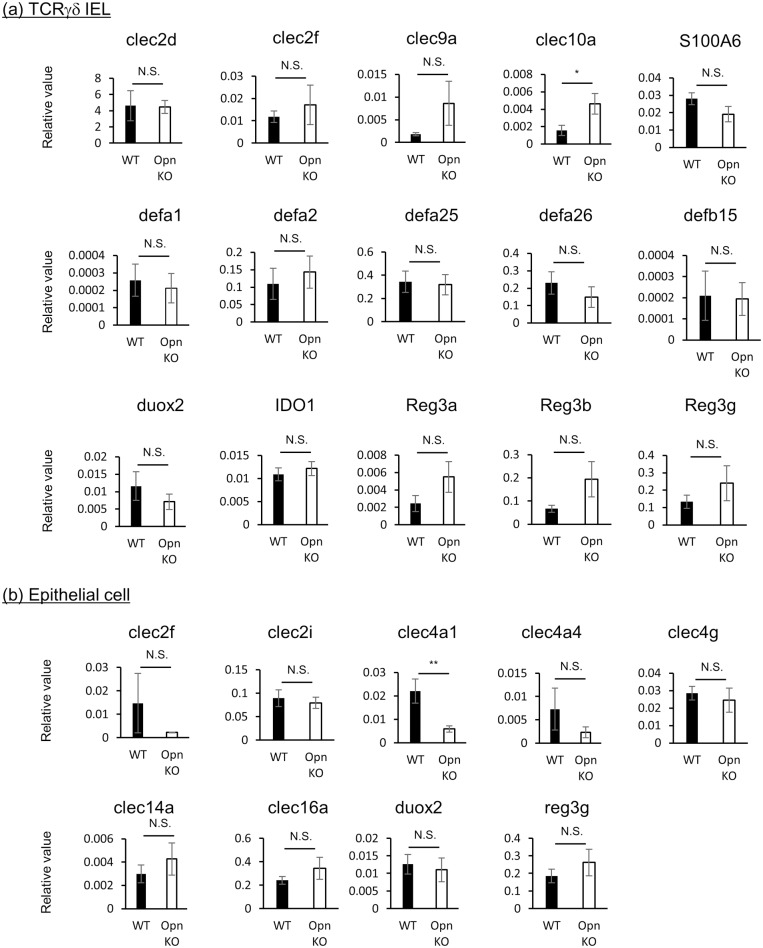
Comparison of antimicrobial factor gene expression in TCRγδ^+^ IELs and epithelial cells from Opn KO and WT mice. Quantitative PCR analysis of antimicrobial factors in (a) TCRγδ IELs and (b) epithelial cells from Opn KO and WT mice. Bars indicate means ±S.E.M. (n = 4). Data is representative of two independent experiments.

Finally, we examined the expression profile of antimicrobial proteins in Opn KO mice. To this end, we sorted CD8α^+^TCRγδ^+^IELs and epithelial cells (CD103^-^EpCAM^+^) from Opn KO or WT mice (sorting schema and purity achieved are shown in S3 Fig), and performed quantitative real-time PCR analysis (Fig 7). In IELs from Opn KO mice, the expression of Clec10a was upregulated (Fig 7a), while in epithelial cells from Opn KO mice, the expression of Clec4a1 was downregulated (Fig 7b) compared to WT mice. However, the expression of most genes was found to be comparable between WT and Opn KO mice.

## Discussion

In this study, we examined the role of Opn in the normal intestine. To explore the source of Opn, we analyzed EGFP-Opn knock-in (KI) mice [26]. In a previous study, it has been suggested that Opn is distributed on epithelial cells and plasma cells [9]. However, we found that epithelial cells and plasma cells were not major sources of Opn (S1 Fig). Moreover, using KI mice, we found that CD8α T cells residing in intestinal tissue, including PP, LP, and IEL, express Opn (Fig 1a). Further, of the cells in the IEL, CD8α T cells showed the highest expression of Opn (Fig 1a). Previously, it was reported that Opn expression on T cells is found in *ex vivo* TCR-stimulated cells and aged mice, involving IFN-γ expression and spontaneous GC formation, respectively [26,28]. IFN-γ protects from colitis via upregulation of MHC class II on intestinal epithelial cells [33] and GC formation in PPs is critical for IgA production and subsequent maintenance of commensal bacteria [34]. Although TCRγδ^+^ IELs are known to be critical for the maintenance of microflora and prevent Salmonella infection via production of antimicrobial peptides [17], the contribution of Opn in TCRγδ^+^ IELs remains unclear. These issues collectively lead us to examine the role of Opn-expressing T cells in normal intestinal tissue. Interestingly, Opn KO mice had altered bacterial communities in their feces (Fig 3). It has been demonstrated that PD-1^-/-^ mice, which exhibit an increased number of follicular helper T cells with a dysfunctional phenotype, have altered microflora as a con of production of dysfunctional IgA [25]. PPs are the main inductive site of IgA [34] and GC formation occurs spontaneously in response to gut bacteria. Specifically, follicular helper T cells are recruited into the PP B cell follicle and interact with B cells, resulting in class switch recombination to IgA production due to cytokine conditions. In a recent report, Tahir *et al*., demonstrated that Opn-producing CD153^+^PD-1^+^CD44^hi^ CD4 T cells promote spontaneous GC formation in the spleen of lupus-prone New Zealand Black x White F1 mice [26]. In addition, a small fraction of CD8 T cells contributes to the generation of IgA-producing B cells [35]. Nevertheless, we observed that deficiency in Opn did not reduce GC formation or IgA production in the PP (Fig 4a and 4b) or binding of IgA to fecal bacteria (Fig 4c). These data demonstrate that Opn production is dispensable for GC formation as well as IgA production and function. In contrast to what has been reported for Opn-expressing CD4 T cells [26], intestinal Opn-expressing CD8 T cells were found to reside at a location distal to the B cell area (Fig 2a). Thus, it is likely that the anatomical distribution of Opn-expressing CD8 T cells is the causative factor for their failure to affect GC formation and IgA production.

In this study, we found that the frequency and number of CD8α^+^TCRγδ^+^ IELs was reduced in Opn KO mice (Fig 5). Opn is known to be a survival factor for activated T cells in the central nervous systems of mice with EAE [32]. Consistent with this report, when we examined the survival of IELs using anti-Opn blocking antibody and Opn KO mice, loss of Opn function resulted in the inhibition of IEL survival (Fig 6), suggesting that Opn is involved in IEL survival. In addition, there is a reduction in the frequency and number of TCRγδ^+^ cells, but not in TCRαβ^+^ cells (Fig 5). These data collectively suggest that the contribution of Opn to cell survival is higher in TCRγδ^+^ IELs *in vivo*. However, in our study, the inhibitory effect on IEL survival was only partial when we used anti-Opn antibody or in Opn KO mice. IL-7 and IL-15 are critical factors for survival of TCRγδ^+^ IEL cells [36–38]. Of note, Cantor et al., recently reported that intracellular Opn (iOpn) promotes the ability of IL-15Rα to bind its ligand, resulting in the prolonged survival of NK cells [39]. This report suggests the possibility that iOpn also contributes to the survival of TCRγδ^+^ IELs, and thus, we will need further investigation of the mechanisms underlying Opn-mediated survival of TCRγδ^+^ IELs.

Antimicrobial peptides are produced from TCRγδ^+^ IELs and intestinal epithelium, resulting in the regulation of microflora. Therefore, we finally examined whether expression of antimicrobial factors by CD8α^+^ TCRγδ^+^ IELs and epithelial cells is affected by the expression of Opn. In IELs and epithelial cells, almost all of the antimicrobial factors that we examined were found to be comparable between WT and Opn KO mice, with the exception of CLEC10a and CLEC4a1 (Fig 7). Thus, Opn has little effect on the expression of antimicrobial factors in the intestinal tissue.

Here we propose a unique function of Opn in the regulation of intestinal homeostasis. As illustrated in S4 Fig, TCRγδ^+^ IELs express Opn. Moreover, Opn expression, possibly both of the secreted form and the intracellular form, increases survival of these cells. In Opn KO mice, TCRγδ^+^ IELs are reduced due to a failure to survive compared to their WT counterparts. As a con, the amounts of antimicrobial factors produced are reduced, resulting in the subsequent alteration of the microflora. However, in this study, we compared fecal microbiota from Opn KO mice and WT mice purchased from different suppliers and maintained for at least three generations in same facility. As microbiota are largely dependent on the nursing mother [40], cohousing of adult mice as performed in this study may not be sufficient to insure uniformity of microbiota. Thus, although our data suggest that Opn is involved in the regulation of microbiota, further investigation using littermates will be required in the future to confirm this.

It has been demonstrated that changes in the microbiota composition are associated with allergy and inflammatory/autoimmune diseases [41]. For instance, it has been reported that obese patients show higher ratios of *Firmicutes* / *Bacteroidetes* than healthy individuals [42]. In the current study, in Opn-deficient mice *Firumicutes* were increased while *Bacteroidetes* were decreased. Interestingly, a recent paper shows an increased ratio of fat to body weight in Opn deficient mice [43]. Collectively, our data suggest Opn might be involved in the regulation of gut microflora via maintenance of TCRγδ^+^ IELs, and therefore, may be implicated in obesity and associated inflammatory diseases and further study of Opn in this context is warranted.

## Supporting information

S1 Fig Expression of Opn in plasma cells and epithelial cells. GFP expression in plasma cells and epithelial cells (B220^-^CD138^+^CD38^+^ and EpCAM^+^CD103^-^, respectively) from intestinal epithelial tissues (Int) and spleens (SPL). Histograms are a representative of three mice. The graph shows the mean of frequency of GFP^+^ plasma cells, epithelial cells, or CD8α cells. Bars indicates ±S.E.M (n = 3, per group). Data are representative of two independent experiments.
(PDF)

S2 Fig Most GFP^+^ TCRγδ cells in the IEL do not express CD8β. The expression of CD8β on CD8α^+^ IEL or splenocytes was analyzed using eight-week-old female KI mice. IELs and cells obtained from spleens were stained with CD3, CD8α, TCRγδ, TCRβ, and CD8β. (Upper panels) Histograms showing CD8β expression on GFP^-^ TCRαβ, GFP^-^ TCRγδ, GFP^+^ TCRαβ, and GFP^+^ TCRγδ or splenic CD8α^+^ cells. (Lower panel) The graph shows the mean of frequency of CD8β^+^ cells. Bars indicate ±S.E.M (n = 3, per group). Data are representative of three independent experiments.
(PDF)

S3 Fig Sorting schema of TCRγδ and TCRαβ IEL and intestinal epithelial cells. TCRγδ or TCRαβ IELs were sorted by gating on CD3^+^CD8α^+^TCRγδ or CD3^+^CD8α^+^TCRαβ cells. Intestinal epithelial cells were sorted by gating on EpCAM^+^CD103^-^ cells. Data are representative of 8 mice from two independent experiments.
(PDF)

S4 Fig Schematic diagram of the possible role of Opn in the regulation of intestinal microflora. (Left) In the normal intestine of WT mice, TCRγδ^+^CD8 T cells express Opn. Intracellular Opn contributes to the survival of these cells. TCRγδ^+^CD8 T cells also express various types of antimicrobial factors resulting in the regulation of intestinal microflora. (Right) In Opn KO mice, IEL TCRγδ^+^CD8 T cells were decreased due to a lack of Opn-mediated survival signals. As a consequence, the total amounts of antimicrobial factors were reduced, resulting in the alteration of the microflora.
(PDF)

S1 Table Primers used in this study. (PDF)

S2 Table Bacterial profiles of fecal sample from Opn KO and WT mice. The means (±S.E.M) of the data obtained in Fig 3 using Illumina MiSeq.
(PDF)
