# Supplementary material for: The potential role of Osteopontin in the maintenance of commensal bacteria homeostasis in the intestine
Source: PLoS One. 2017 Mar 15;12(3):e0173629. doi: 10.1371/journal.pone.0173629 (PMC5351998; doi:10.1371/journal.pone.0173629)
Supplement: S1 Fig — GFP expression in plasma cells and epithelial cells (B220-CD138+CD38+ and EpCAM+CD103-, respectively) from intestinal epithelial tissues (Int) and spleens (SPL). Histograms are a representative of three mice. The graph shows the mean of frequency of GFP+ plasma cells, epithelial cells, or CD8α cells. Bars indicates ±S.E.M (n = 3, per group). Data are representative of two independent experiments. (PDF) [file pone.0173629.s001.pdf]

Supplementary information

S 1 Fig. Expression of Opn in plasma cells and epithelial cells

GFP expression in plasma cells and epithelial cells ($B220^{-}CD138^{+}CD38^{+}$ and $EpCAM^{+}CD103^{-}$, respectively) from intestinal epithelial tissues (Int) and spleens (SPL). Histograms are a representative of three mice. The graph shows the mean of frequency of GFP^{+} plasma cells, epithelial cells, or CD8α cells. Bars indicates ±S.E.M (n=3, per group). Data are representative of two independent experiments.

S1 Fig

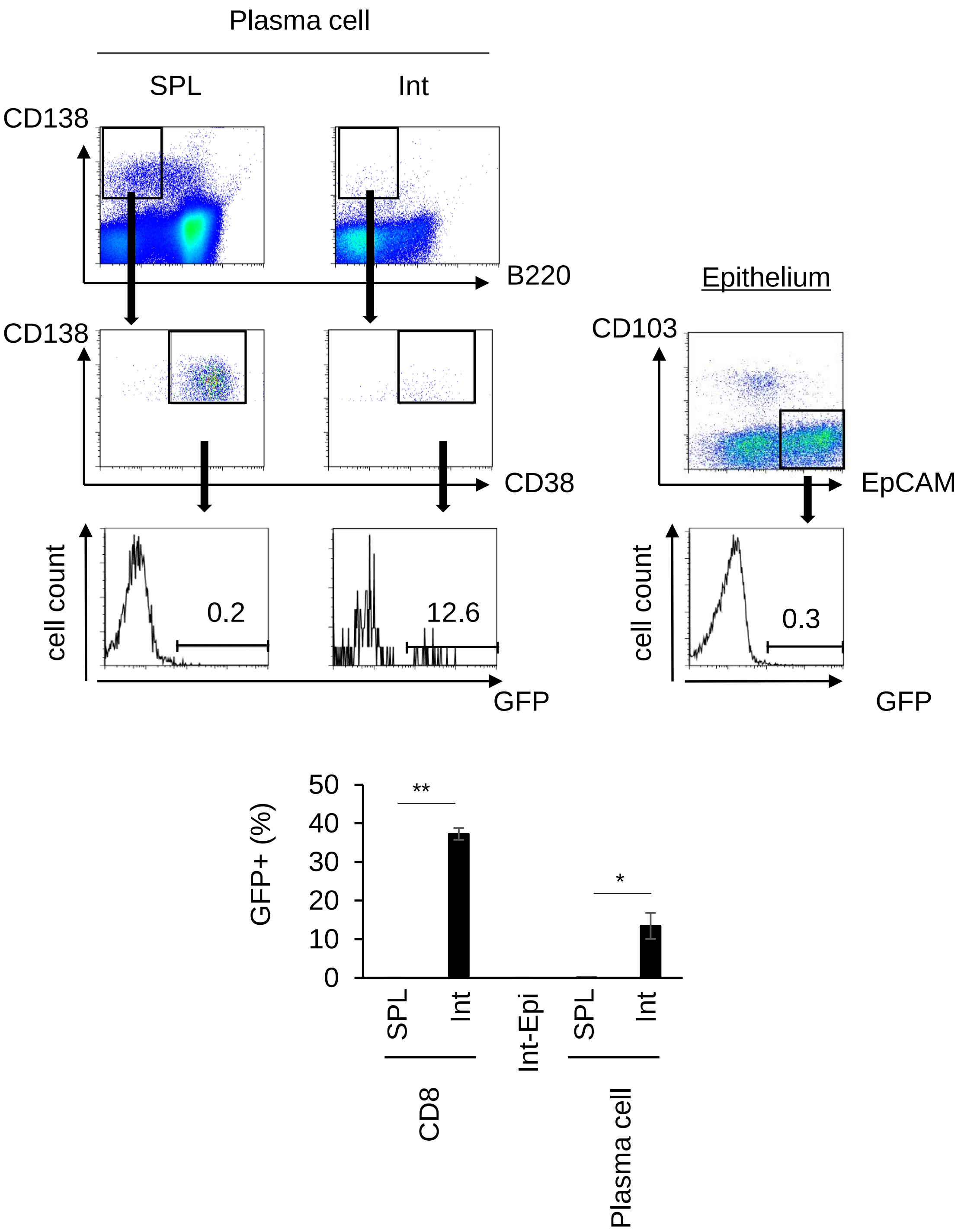

Plasma cell
SPL
Int
CD138
B220
CD138
CD38
Epithelium
CD103
EpCAM
cell count
0.2
12.6
GFP
cell count
0.3
GFP
GFP+ (%)
50
40
30
20
10
0
**
*
SPL
Int
Int-Epi
SPL
Int
CD8
Plasma cell
