# Supplementary material for: The potential role of Osteopontin in the maintenance of commensal bacteria homeostasis in the intestine
Source: PLoS One. 2017 Mar 15;12(3):e0173629. doi: 10.1371/journal.pone.0173629 (PMC5351998; doi:10.1371/journal.pone.0173629)
Supplement: S2 Fig — The expression of CD8β on CD8α+ IEL or splenocytes was analyzed using eight-week-old female KI mice. IELs and cells obtained from spleens were stained with CD3, CD8α, TCRγδ, TCRβ, and CD8β. (Upper panels) Histograms showing CD8β expression on GFP- TCRαβ, GFP- TCRγδ, GFP+ TCRαβ, and GFP+ TCRγδ or splenic CD8α+ cells. (Lower panel) The graph shows the mean of frequency of CD8β+ cells. Bars indicate ±S.E.M (n = 3, per group). Data are representative of three independent experiments. (PDF) [file pone.0173629.s002.pdf]

S 2 Fig. Most GFP$^+$ TCRγδ cells in the IEL do not express CD8β

The expression of CD8β on CD8α$^+$ IEL or splenocytes was analyzed using eight-week-old female KI mice. IELs and cells obtained from spleens were stained with CD3, CD8α, TCRγδ, TCRβ, and CD8β. (Upper panels) Histograms showing CD8β expression on GFP$^-$ TCRαβ, GFP$^-$ TCRγδ, GFP$^+$ TCRαβ, and GFP$^+$ TCRγδ or splenic CD8α$^+$ cells. (Lower panel) The graph shows the mean of frequency of CD8β$^+$ cells. Bars indicate ±S.E.M (n=3, per group). Data are representative of three independent experiments.

S2 Fig

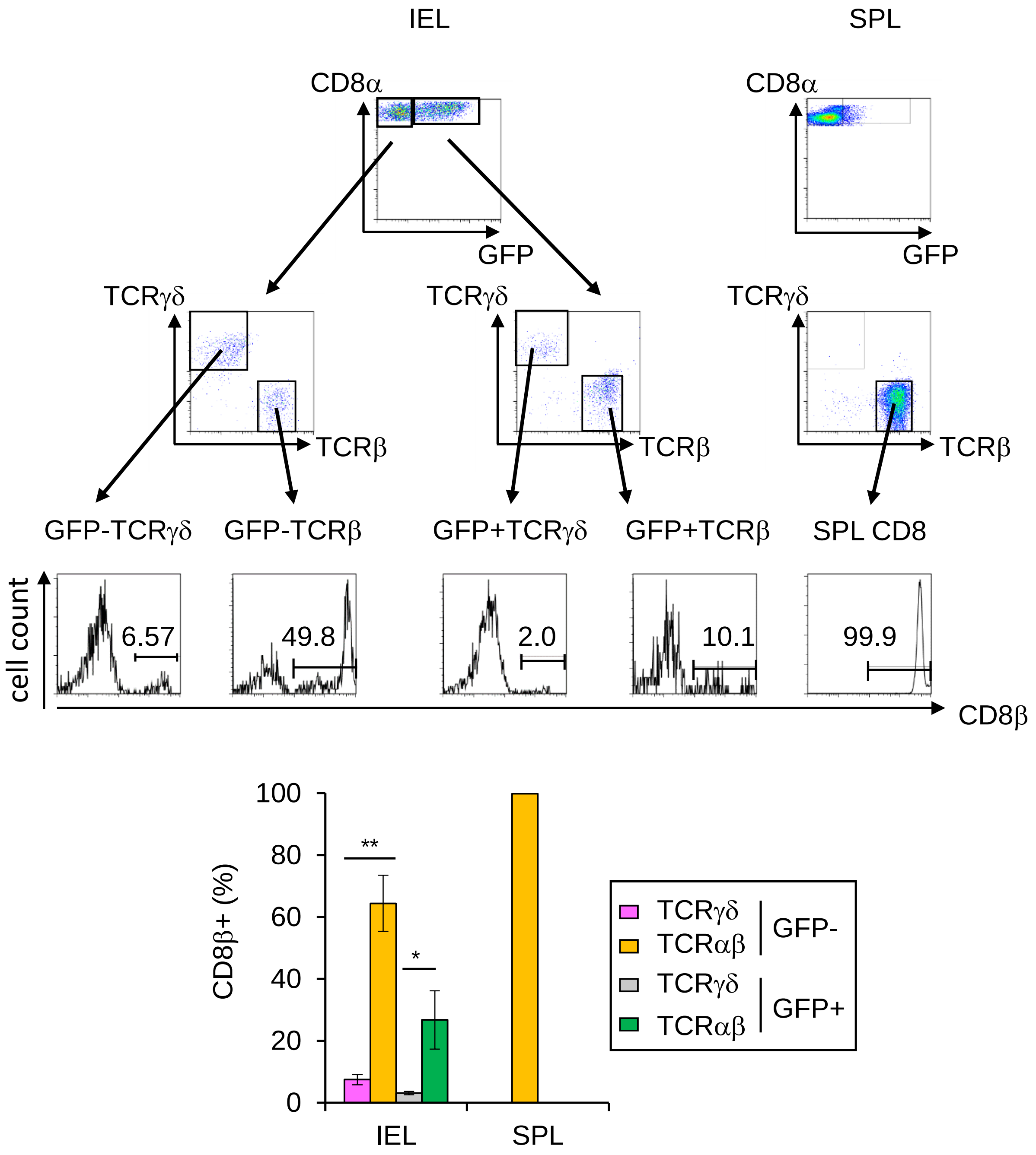

IEL
SPL
CD8α
GFP
CD8α
GFP
TCRγδ
TCRβ
TCRγδ
TCRβ
TCRγδ
TCRβ
GFP-TCRγδ
GFP-TCRβ
GFP+TCRγδ
GFP+TCRβ
SPL CD8
cell count
6.57
49.8
2.0
10.1
99.9
CD8β
100
80
60
40
20
0
CD8β+ (%)
**
*
IEL
SPL
TCRγδ
TCRαβ
GFP-
TCRγδ
TCRαβ
GFP+
