# Supplementary material for: The potential role of Osteopontin in the maintenance of commensal bacteria homeostasis in the intestine
Source: PLoS One. 2017 Mar 15;12(3):e0173629. doi: 10.1371/journal.pone.0173629 (PMC5351998; doi:10.1371/journal.pone.0173629)
Supplement: S3 Fig — TCRγδ or TCRαβ IELs were sorted by gating on CD3+CD8α+TCRγδ or CD3+CD8α+TCRαβ cells. Intestinal epithelial cells were sorted by gating on EpCAM+CD103- cells. Data are representative of 8 mice from two independent experiments. (PDF) [file pone.0173629.s003.pdf]

S3 Fig. Sorting schema of TCRγδ and TCRαβ IEL, and intestinal epithelial cells

TCRγδ or TCRαβ IELs were sorted by gating on $CD3^+CD8\alpha^+$TCRγδ or $CD3^+CD8\alpha^+$TCRαβ cells. Intestinal epithelial cells were sorted by gating on $EpCAM^+CD103^-$ cells. Data are representative of 8 mice from two independent experiments.

S3 Fig

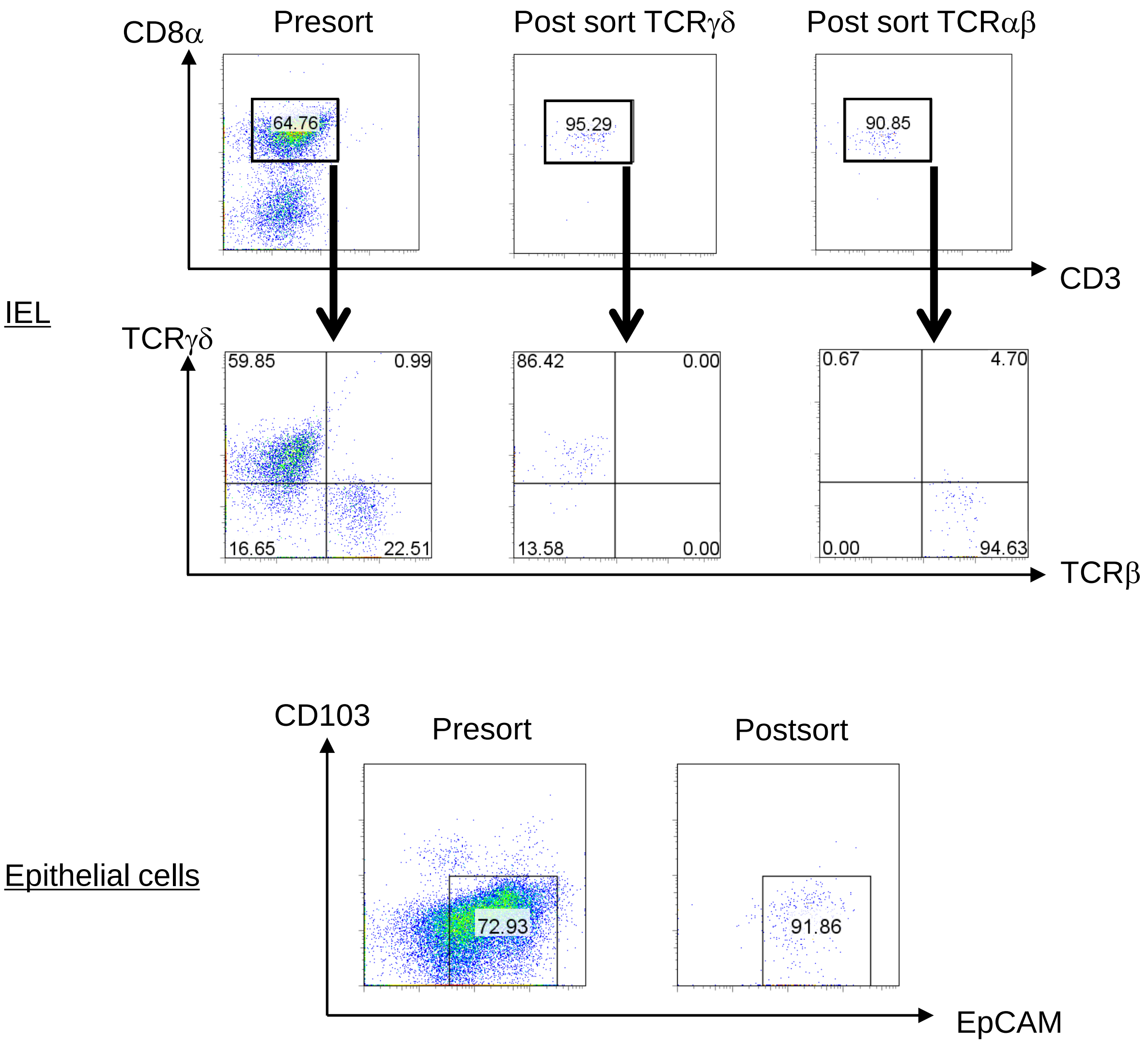
CD8α
Presort
Post sort TCRγδ
Post sort TCRαβ
64.76
95.29
90.85
CD3
IEL
TCRγδ
59.85
0.99
16.65
22.51
86.42
0.00
13.58
0.00
0.67
4.70
0.00
94.63
TCRβ
CD103
Presort
Postsort
Epithelial cells
72.93
91.86
EpCAM
