# Supplementary material for: The potential role of Osteopontin in the maintenance of commensal bacteria homeostasis in the intestine
Source: PLoS One. 2017 Mar 15;12(3):e0173629. doi: 10.1371/journal.pone.0173629 (PMC5351998; doi:10.1371/journal.pone.0173629)
Supplement: S4 Fig — (Left) In the normal intestine of WT mice, TCRγδ+CD8 T cells express Opn. Intracellular Opn contributes to the survival of these cells. TCRγδ+CD8 T cells also express various types of antimicrobial factors resulting in the regulation of intestinal microflora. (Right) In Opn KO mice, IEL TCRγδ+CD8 T cells were decreased due to a lack of Opn-mediated survival signals. As a consequence, the total amounts of antimicrobial factors were reduced, resulting in the alteration of the microflora. (PDF) [file pone.0173629.s004.pdf]

S4 Fig. Schematic diagram of the possible role of Opn in the regulation of intestinal microflora

(Left) In the normal intestine of WT mice, TCRγδ^+CD8 T cells express Opn. Intracellular Opn contributes to the survival of these cells. TCRγδ^+CD8 T cells also express various types of antimicrobial factors resulting in the regulation of intestinal microflora. (Right) In Opn KO mice, IEL TCRγδ^+CD8 T cells were decreased due to a lack of Opn-mediated survival signals. As a consequence, the total amounts of antimicrobial factors were reduced, resulting in the alteration of the microflora.

S4 Fig

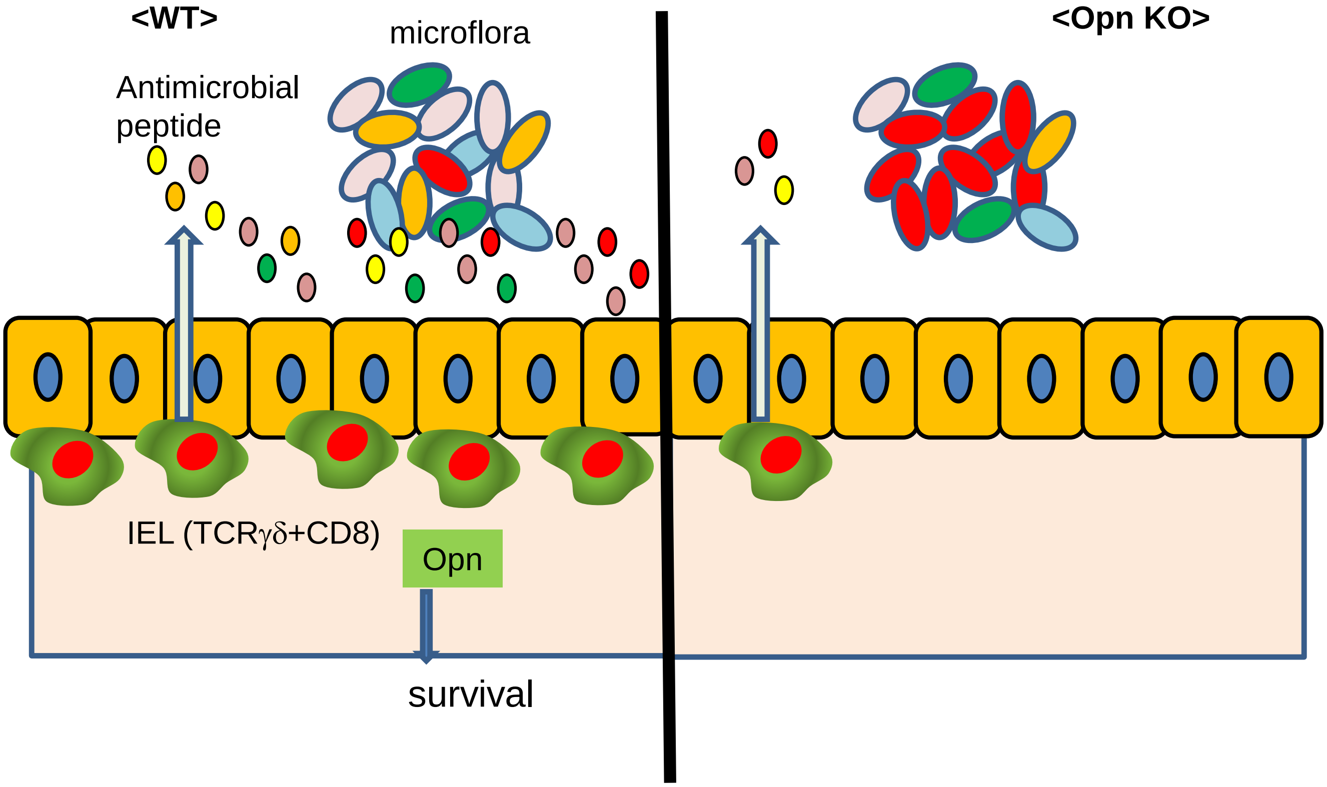

<WT>
microflora
<Opn KO>
Antimicrobial
peptide
IEL (TCRγδ+CD8)
Opn
survival
