# Supplementary material for: The potential role of Osteopontin in the maintenance of commensal bacteria homeostasis in the intestine
Source: PLoS One. 2017 Mar 15;12(3):e0173629. doi: 10.1371/journal.pone.0173629 (PMC5351998; doi:10.1371/journal.pone.0173629)
Supplement: S1 Table — (PDF) [file pone.0173629.s005.pdf]

S1Table. Primers used in this study

S1 Table Primers used in this study

Genes			Genes		
defb15	Fw	5’-ttgctgttctcttctttctgga-3’	IDO1	Fw	5’-gggctttgctctaccacatc-3’
	Rv	5’-ctgtcacacagcacttcagattc’-3’		Rv	5’-aaggacccaggggctgtat-3’
clec16a	Fw	5’-ggcacagaaagtcatcctcag-3’	Reg3g	Fw	5’-accatcaccatcatgtcctg-3’
	Rv	5’-agttgggcatccacttgc-3’		Rv	5’-ggcatctttcttggcaactt-3’
clec2d	Fw	5’-cagacaacactgagtataacaacacg-3’	Reg3b	Fw	5’-tggattgggctccatgac-3’
	Rv	5’-cccgttgttgttcaggtagg-3’		Rv	5’-tcatcacgtcattgttactcca-3’
defa1	Fw	5’-ctttgcccttgtcctgctt-3’	clec10a	Fw	5’-agaaaacccaagagcctggt-3’
	Rv	5’-ttcatctgtgttttggatagaatca-3’		Rv	5’-gaggcccagggagaacag-3’
duox2	Fw	5’-gcgctctgctgactggac-3’	clec2f	Fw	5’-cccaactagctcggtttgac-3’
	Rv	5’-attaaaccagccgtcgtagc-3’		Rv	5’-ccagtggctagaattcatgtgtc-3’
defa2	Fw	5’-ctgcccttgtcctactgtcc-3’	clec4g	Fw	5’-ctaccggtgggtagatggag-3’
	Rv	5’-ctcttcatctgtgttttggatagg-3’		Rv	5’-tctggaatcattgggctctc-3’
defa25	Fw	5’-agggaaagaagaccaagctgt-3’	clec4a1	Fw	5’-tcatctgctggtgatccaga-3’
	Rv	5’-ttttgcagcctcttgttctaca-3’		Rv	5’-ataagcagcacgagggttca-3’
defa26	Fw	5’-aggtgcagccacaggaag-3’	clec4a4	Fw	5’-gggtgctcatctggtggt-3’
	Rv	5’-catcccagatctctcaacgatt-3’		Rv	5’-accagcacttgtgttcaggtt-3’
Reg3a	Fw	5’-attgggctccatgatcca-3’	clec14a	Fw	5’-gaaagagacacttgttccctgaa-3’
	Rv	5’-agataattcagcacatcggagtt-3’		Rv	5’-cttccaataccagcccaca-3’
S100A6	Fw	5’-ctgcccttgtcctactgtcc-3’	clec2i	Fw	5’-catgccagattgcttgga-3’
	Rv	5’-ctcttcatctgtgttttggatagg-3’		Rv	5’-tctgtacacactgggcattca-3’
clec9a	Fw	5’-ggcttgttagcaacgtccat-3’			
	Rv	5’-ctgctccaagacaagagagga-3’			
