# Supplementary material for: The potential role of Osteopontin in the maintenance of commensal bacteria homeostasis in the intestine
Source: PLoS One. 2017 Mar 15;12(3):e0173629. doi: 10.1371/journal.pone.0173629 (PMC5351998; doi:10.1371/journal.pone.0173629)
Supplement: S2 Table — The means (±S.E.M) of the data obtained in Fig 3 using Illumina MiSeq. (PDF) [file pone.0173629.s006.pdf]

S 2 Table. Bacterial profiles of fecal samples from Opn KO and WT mice

The means (±S.E.M) of the data obtained in Fig. 3 using Illumina MiSeq.

S2 Table Bacterial profiles of fecal sample from Opn KO and WT mice

Bacteria (Phyla)	WT (count±S.E.M)	Opn KO (count±S.E.M)	P-value
Actinobacteria	282.2± 184.8	104.2 ± 94.7	0.21
Bacteroidetes	49500.2 ± 3092.8	38587.2 ± 4731.1	0.04*
Cyanobacteria	320.2 ± 72.7	298 ± 43.9	0.4
Defferibacteres	291.4 ± 90.8	1600.4 ± 524.2	0.019*
Firmicutes	13969.8 ± 3357.2	24107.2 ± 4536.7	0.055
Proteobacteria	1964.6 ± 238.2	3337.4 ± 669.6	0.044*
Tenericutes	68.6 ± 12.7	298.6 ± 111.0	0.036*
Verrucomicrobia	459.6 ± 307.4	25.2 ± 16.7	0.097
